# Trade-offs between voice and silence: a qualitative exploration of oncology staff’s decisions to speak up about safety concerns

**DOI:** 10.1186/1472-6963-14-303

**Published:** 2014-07-14

**Authors:** David LB Schwappach, Katrin Gehring

**Affiliations:** 1 Swiss Patient Safety Foundation, Asylstr. 77, 8032 Zuerich, Switzerland; 2 Institute of Social and Preventive Medicine (ISPM), University of Bern, Bern, Switzerland

**Keywords:** Communication, Safety culture, Qualitative research, Patient safety, Oncology

## Abstract

**Background:**

Research suggests that “silence”, i.e., not voicing safety concerns, is common among health care professionals (HCPs). Speaking up about patient safety is vital to avoid errors reaching the patient and thus to prevent harm and also to improve a culture of teamwork and safety. The aim of our study was to explore factors that affect oncology staff’s decision to voice safety concerns or to remain silent and to describe the trade-offs they make.

**Methods:**

In a qualitative interview study with 32 doctors and nurses from 7 oncology units we investigated motivations and barriers to speaking up towards co-workers and supervisors. An inductive thematic content analysis framework was applied to the transcripts. Based on the individual experiences of participants, we conceptualize the choice to voice concerns and the trade-offs involved.

**Results:**

Preventing patients from serious harm constitutes a strong motivation to speaking up but competes with anticipated negative outcomes. Decisions whether and how to voice concerns involved complex considerations and trade-offs. Many respondents reflected on whether the level of risk for a patient “justifies” the costs of speaking up. Various barriers for voicing concerns were reported, e.g., damaging relationships. Contextual factors, such as the presence of patients and co-workers in the alarming situation, affect the likelihood of anticipated negative outcomes. Speaking up to well-known co-workers was described as considerably easier whereas “not knowing the actor well” increases risks and potential costs of speaking up.

**Conclusions:**

While doctors and nurses felt strong obligation to prevent errors reaching individual patients, they were not engaged in voicing concerns beyond this immediacy. Our results offer in-depth insight into fears and conditions conducive of silence and voicing and can be used for educational interventions and leader reinforcement.

## Background

Communication breakdowns among healthcare professionals (HCPs) have been identified as a major threat to patient safety and to the prevention of errors [1]. Speaking up about patient safety is vital to avoid errors reaching the patient and thus to prevent harm and also to improve a culture of teamwork and safety. “Speaking up” can be defined as assertive communication in clinical situations that require (immediate) action through questions or statements of opinion or information with appropriate persistence until there is a clear resolution [2,3]. This type of problem-focused voice can be distinguished from suggestion-focused and opinion-focused types of voice [4]. “Silence”, i.e., not voicing safety concerns, however, is a common behaviour among health care professionals (HCPs). The “Silence Kills” study revealed that 85% of nurses had been warned of a problem by a safety tool, e.g., the surgical safety checklist, but 58% had also been in situations where they felt it was unsafe to speak up to their colleagues [5]. St. Pierre et al. conducted a full-scale simulation study in anesthesiology to observe speaking up behaviors. Nurses and residents challenged an attending in only few of the critical situations created by the attendant, including fatal drug administrations [6]. Speaking up behaviour of nurses has been associated with improved team performance of operating room teams in a simulation study [7].

Little is known about factors that determine HCPs’ willingness to speak up for safety. In interdisciplinary action teams, e.g., in operation rooms, two barriers have been identified that inhibit speaking up: Power discrepancies, e.g., between nurses and surgeon, and staff’s lack of conviction that their input is needed and desired, i.e., little motivation that makes the effort and risk worthwhile [8]. In a qualitative study in midwifery, agency for safety (the willingness to take a stand on an issue of concern) fluctuated strongly in relation to context and social relationships between involved health care professionals [9]. Novelty of an alarming situation and the fear to damage personal relationship inhibited agency for safety. In a survey among US and Japanese residents, the decision to challenge a senior surgeon in the operating room was affected by the relationship and anticipated response of the superior [10]. Lyndon et al. conducted a quantitative vignette survey about speaking up about safety concerns in labour and delivery [3]. Potential for harm ratings were the strongest single predictor for likelihood of speaking up. Among all respondents, 12% were reluctant to speak up despite perceiving a high potential for harm to the patient. The association of harm rating and likelihood of speaking up was attenuated by the hierarchy of the professional involved in the case scenario. From this preliminary research it can be concluded that assessments of harm seem to play an important role in HCPs’ speaking up behaviours and that these assessments may differ between professional roles.

Research on organizational silence outside the healthcare setting suggests that decisions to speak up are strongly influenced by organizational context and their interaction effects with individual-level factors [11]. Morrison and Milliken define a climate of silence as one characterized by two shared beliefs: (1) speaking up about problems is not worth the efforts, and (2) speaking up is dangerous [12]. They developed a model in which the choice to remain silent is driven by individual characteristics, organizational characteristics, relationship with the supervisor, anticipated negative outcomes, and the belief that speaking up will not make a difference [13]. Detert and Edmonson conducted research about improvement-oriented voicing in organizations [14]. The results confirm that employees have implicit theories about leaders’ reactions to speaking up and that these beliefs, which may or may not be accurate, limit willingness to speak up. Opportunities for voicing concerns seem to be evaluated for risk episodically and the range of perceived risk of speaking up is considerable. As Okuyama et al. emphasize, health care differs from other industries in that speaking up for safety is primarily aimed at promoting patients’ wellbeing and not that of the organization or the self [15]. Preventing patients from expected and serious harm constitutes a strong and immediate motivation to speak up but competes with anticipated negative outcomes and fears about personal consequences. Clearly, HCPs make some trade-off when they decide whether to speak up or not but there has been little research so far into the factors that enter and constitute this trade-off in health care in particular.

Most of the empirical evidence related to speaking up in health care has been conducted in invasive care (surgery, anaesthesiology) and in midwifery. There is a paucity of research in other areas of medicine. In oncology, a multiprofessional and complex area of health care, in which single small errors may result in serious patient harm, effective and assertive communication about errors and risky behaviours between team members is essential. In cancer care, a variety of situations could warrant speaking up including medication safety concerns, in particular prescription and administration errors or violations of safety tools and protocols for safe medication practices, violations of hospital hygiene rules (e.g., for patients in isolation), safety in the context of invasive procedures (e.g., lumbar puncture, catheters, ports), and also treatment decisions. Yet, nothing is known about oncology staff’s speaking up behaviours. Patients are very vulnerable, on both somatic and psychological dimensions, and professionals are typically highly engaged in building and preserving a trustful relationship with patients. This may inhibit open communication about close-calls or safety rule violations and add to the trade-off whether and how to express safety concerns. At least when patients are observers of HCPs speaking up behaviours, “promoting the patient’s wellbeing” may be a catalyst for both, voice and silence. For example, a nurse may feel that challenging a senior doctor about hand disinfection in front of a patient would threaten the trustful doctor-patient-relationship which outweighs the risk of infection and consequently decide to withhold her voice. The aim of our study was to explore factors that affect oncology staff’s decision to voice safety concerns or to remain silent. We address two research gaps: The first concerns limited knowledge about speaking up behaviour in oncology, a special area of clinical care for the reasons outlined above. The second gap results from a lack of theory in models of speaking up in health care. In a qualitative interview study with doctors and nurses from oncology units we investigated reasons, facilitators and barriers to speaking up towards co-workers and supervisors. We conducted semi-structured interviews rather than a quantitative survey to be able to gain a deeper understanding of the factors affecting deliberate decisions to speak up rather than using predefined categories of behaviour. We wanted to gain knowledge about how HCPs arrive at their decisions to voice concerns, something that is difficult to assess in a survey. Our interview guide was inspired by theoretical models of speaking up decisions by Milliken et al. and Morrison [4,13]. These general models of voicing behaviour have recently been modified and adapted to the health care setting [15]. We used thematic content analysis to identify common themes and dominant arguments. Thematic content analysis is a qualitative descriptive methodology which requires a lower level of interpretive complexity, as compared to grounded theory or phenomenology and thus seems adequate for a research question conducted within a theoretical framework [16]. Based on the individual experiences of HCPs from different professional groups and of different hierarchical status, we aimed to conceptualize the choice to voice concerns and the trade-offs involved and thereby to contribute to the theoretical refinement of existing models.

## Methods

### Interviews

We conducted semi-structured interviews with experienced oncology staff. The topic guide was developed based on prior research, the literature, and discussions with oncology experts [13,17-19]. The interviews began with general questions about how often participants experience situations that they felt would require voicing their concern and whether they generally feel comfortable to raise patient safety issues with co-workers and supervisors in their team. The central part of the interview asked participants about situations where they experienced concerns about patient safety and decided whether and how to communicate them. Participants were inquired to describe these situations and the contextual factors surrounding them. We asked respondents about the reasons and motivations (drivers, ambitions) underlying their decisions to voice concerns or remain silent. They were encouraged to elaborate on facilitators and barriers affecting their choices for articulating safety concerns. We did not explicitly ask for “trade-offs”, “anticipations”, “calculations” or similar concepts nor did we use these terms.

### Sample

Six hospitals participated with seven oncology departments in Switzerland. These included three regional hospitals and two university hospitals with adult oncology units, and two paediatric university hospital departments. Nurses and doctors working at the oncology departments of the participating hospitals received written study information and were invited to register for an interview. Interviewees were purposively sampled to include doctors and nurses, staff working at the ambulatory oncology units or on ward, and with sufficient working experience in oncology. Interviewees gave prior written informed consent. Interviews were conducted face-to-face at the hospitals by a trained and experienced research assistant, audio-recorded and transcribed verbatim. The study was exempted from full ethical review by the Cantonal Ethics Committee (KEK-StV-Nr. 58/13).

### Data analysis

An inductive thematic content analysis framework was applied to the transcripts [20-23]. After listening to interviews and reading the transcripts to become familiar and get a sense of the whole, two researchers independently analysed and coded a subset of transcripts (open coding) using a mixed-methods research software [24]. The texts were excerpted into units of meaning (words, sentences or paragraphs). Emergent themes and recurring ideas were identified and classified in terms of the concepts arising from it. The code structure was discussed and revised and applied to the next subset of transcripts in an iterative process. Again, areas of disagreement were discussed in feedback-loops to increase validity [22,25]. New codes were added as additional themes emerged and some codes were eliminated. The finalized code structure was then applied to all transcripts by both researchers, and any discrepancies were solved [21,26]. Categories were abstracted as far as possible by grouping sub-categories as categories and categories as themes [22]. For the purpose of this analysis, we concentrate on content related to the decision to communicate safety concerns. Data were organized in major themes relevant for the research question. Representative quotes were selected. Throughout, we use the term “observer” for the professional who detected an error or violation of safety rule (the interviewee) and “actor” for the person who performs the questionable behaviour or to whom the error was attributed to.

## Results

Interviews were conducted with 32 doctors and nurses in oncology. The interviews lasted between 21–58 minutes (mean 42 minutes). Characteristics of the participants are provided in Table 1. Participants reported a broad range of detailed situations where they had experienced safety concerns and decided whether and how to communicate these to their co-workers or supervisors.

**Table 1:** Characteristics of participants

**Characteristic**		**Count**	**%**
Age (median = 35 years, range = 23-62 years)			
	20-35 years	18	56
	36-45 years	6	19
	46-65 years	8	25
Female gender		22	69
Profession/function			
	Head nurse	3	9
	Nurse	15	47
	Resident	10	31
	Senior doctor	4	13
Primary workplace			
	Ambulatory oncology unit	17	53
	Ward	15	47
Hospital type			
	Regional	15	47
	University: Adult oncology	11	34
	University: Pediatric oncology	6	19
Months of work experience in oncology (median = 42 months, range = 2-312 months)			
	1-18 months (<= 1.5 years)	13	41
	19-83 months (1.5 – 7 years)	8	25
	84 months and more (> = 7 years)	11	34

Errors in the context of medication safety and rule violations in hygiene and isolation practices were the most commonly described safety issues which prompted concerns. Participants felt generally comfortable voicing concerns, in particular related to medication safety. However, they provided numerous and diverse reasons for and against speaking up and elaborated how these enter the choice to voice concerns.

### Motivations for speaking up

The predominating, overarching motivation for voicing concerns expressed by the vast majority of participants was to protect patients from injury. All accounts of “preventing harm” referred to individual identifiable patients. Contrary, saving future unknown “lives” (e.g., by improving safety standards in the unit) was not mentioned as motivation for speaking up at all. Other reasons for speaking up were only rarely mentioned. Notably, only two participants explained that they voiced to protect a co-worker from causing harm to a patient, what, in turn would have serious consequences for the colleague (i.e., feelings of guilt and loss of reputation). Three doctors elaborated that speaking up about patient safety would be a proof of their seriousness and would contribute to one’s positive image. Table 2 displays motivations to speak up with example quotes.

**Table 2:** Motivations to speak up

**Motivation**	**Example quotes from interviewees**
Protect patient from harm	• *“Of course…to protect the child from the specialist.“* Resident, pediatric ambulatory unit (L131)
	• *“The point is that nothing serious happens. That is most important to me”.* Nurse, pediatric ambulatory unit (L232)
Contribute to one’s image	• *“I had the feeling that it speaks in my favor. That it demonstrates that I take my job seriously”.* Resident, ambulatory unit (P127)
Protect the actor from causing harm	• *“…to protect the patient, and of course, the nurse, who made the mistake. It is a devastating experience to be responsible for patient harm and I wanted to save her from that”.* Nurse, ambulatory unit (P229)

### Barriers to speaking up

Participants expressed a large variety of barriers to speaking up and conditions and constellations conducive of “silence” (Table 3 displays categories of barriers to speak up with example quotes). A major barrier to expressing concerns was related to the presence of other persons in the alarming situation. 20 respondents said, sometimes strongly, that avoiding exposure and humiliation of the actor in front of co-workers or patients was the main barrier for voicing concerns.

**Table 3:** Barriers to speak up

**Barrier**	**Example quotes from interviewees**
Presence of other persons	• *“I do not want to humiliate anyone”.* Nurse, ward (C114)
Erosion of trust between patient and caregiver	• *“It is very challenging to speak up directly. There is the patient, and mother or father present. These are very tough situations, where you know you should voice and you want to, and still, it is difficult. You cannot voice your concerns in front of the patient”.* Nurse, pediatric ambulatory unit (L232)
Embarrassment and humiliation of the actor	
	• *“This basic trust of the patient towards doctors, you do not want to shake their confidence. Thus, not in front of the patient, if possible”.* Resident, ward (J118)
Hierarchical structures and relations	• *“Because I have the feeling… that it is just not done. I would be so worried with how to say it to him [the chief of department], that it would be too late then, or I would end up sweating”.* Resident, pediatric ward (J122)
Experience/knowledge gap (observer/actor)	
	• *“I, as a junior doctor, just feel uncomfortable to speak up towards a senior colleague with ages of experience. It…doesn’t feel right. You ask yourself whether these shoes aren’t too large…to challenge someone with lots of experience, knowledge and responsibilities. That’s a problem”.* Resident, ambulatory unit (P126)
Limited time	• *“If you have lots of things to do you think, well okay, it just is like this now”.* Nurse, ambulatory unit (C113)
Speed of the incident	
Observer’s limited time resources	• *“He [the senior] has several ward rounds to do, he is under stress, he is under time pressure. And to hold him back with something like this – difficult”.* Resident, ambulatory unit (B101)
Actor’s time constraints and distress	• *“Sometimes, raising concerns makes me feel guilty because time is so short at the rapport”.* Resident, ambulatory unit (X110)
Fears of negative consequences	• *“Fear, of course. Will I find the right words and tone?”* Nurse, ward (J221)
	• *“..and when you are still in training, challenging the supervisors is not good. Too much confrontation is not good at all”.* Resident, ambulatory unit (P127)
Prompt negative or harsh reactions	
Being labelled as “difficult”	• *“Because she is a special character and beliefs that she does not make errors. When you tell her that she did something wrong she will be touchy for days”.* Resident, pediatric ward (J122)
Damaging good relationships	
Actor’s personality	
Occupational group constellation (observer/actor)	• *“Simply, because he is a surgeon and we are nurses. Not everybody has the courage to speak up to a surgeon”.* Nurse, ward (J221)
	• *“It is not really well received if you as a doctor point them [nurses] to their lapses. They think that we have no idea of these issues, .. removing catheters, check IV lines more closely or positioning of a patient. Thus we should remain silent”.* Senior doctor, ward (B102)
Futility and resignation	• *“If there is a person, and you can say it over and over, and it doesn’t change. That is difficult”.* Nurse, pediatric ambulatory unit (L232)

A common concern raised by more than a third of interviewees had to do with preserving trust of the patient. Many respondents argued that pointing a co-worker to an error or violation of safety rule with the patient present would erode trust of patients and endanger the caregiver-patient relationship. Hierarchical structures and relations make it difficult to speak up for nearly all participants. These expressions were usually accompanied by vague beliefs that speaking up to superiors would be “inappropriate”. Hierarchy was commonly mentioned in conjunction with a knowledge/experience gap between observer and actor. Observers questioned their own interpretation of the incident or the potential harm because the actor was much more experienced and higher in hierarchy. Interviewees of lower hierarchical status (e.g., residents) argued that social norms prohibited them from speaking up to skip-level superiors and they expected their direct supervisors to voice concerns. If these direct supervisors fail to speak up it becomes nearly impossible for residents to express their concerns. Voicing concerns across professional groups was an inconsistent factor in participants’ reports. Some nurses found it easier to raise concerns towards doctors because they were not perceived as their line superiors; others found it difficult to cross professional boundaries, in particular to higher-level superiors.

Limited time was mentioned as a barrier for discussing safety concerns in several contexts: First, participants reported situations where they noticed errors or violations which happened so quickly that they could not intervene in time (e.g., a nurse saw a co-worker using wrong material for a syringe). Notably, in only few of these occasions the observer decided to discuss the issue afterwards, e.g., to prevent the same incident from happening again. Second, respondents mentioned their own limited time resources as reducing their willingness to voice concerns. Finally, lack of time and stress on the part of the actor was frequently mentioned as a reason for silence. Observers did not want to disturb, or to make matters worse by starting discussions, or feared negative responses of the distressed colleague.

One third of participants expressed fears of negative consequences of their speaking up. This mainly covered fears of stimulating immediate (emotional) reactions, being labelled as “difficult” and damaging good relationships. 13 respondents argued that they would not voice their concerns towards specific, individual co-workers or supervisors because these were “difficult persons”. Futility and resignation were also expressed by several respondents. Respondents reported that earlier experiences of speaking up did not have the desired outcome and thus expressing concerns would be pointless. Commonly, these experiences concentrated on co-workers’ lack of compliance with and violations of safety rules.

### Risk assessment and deliberate trade-offs

It was apparent from participants’ reflections that their decisions whether and how to voice safety concerns involved complex considerations and trade-offs. Several interviewees explicitly used the terms “trade-off”, “balance”, “evaluations of risks and benefits”, “weighting”, and “risk assessment” when elaborating on this process. Subthemes and example quotes are displayed in Table 4.

**Table 4:** Trade-offs between voice and silence

**Themes**	**Example quotes from interviewees**
Judging the level of risk	• *“This is a matter of risk assessment. Of course, there is the risk of infection, no doubt. But in this case, the patient was not in a condition which would make an infection dangerous. But, if, for example, she had had no leucocytes and would develop fever and she would have been at high risk for an infection, than it would be a different situation. I do this risk assessment mentally”.* Resident, ward (J118)
	• *“It is a deliberate balance…when to voice, or how important it is, that you’ll have to voice, and when you don’t have to. I believe there is some grey area. But if something is really dangerous for the patient, then I have to speak up”.* Nurse, pediatric ward (L230)
	• *“I evaluate how high the risk is and, with a missed hand disinfection, would not regard it as high enough to say something. Contrary, if somebody would walk into an isolation room without appropriate clothes, I would say ‘Stop’. No doubt”.* Resident, ambulatory unit (B103)
	• *“But, if I see that someone gives the wrong IV to a patient, I would react immediately. Or if Inotice that the premedication was not given with antibodies and there is the risk that the patient reacts, than I would instantly intervene”.* Nurse, ward (C114)
Differing perceptions of harm between professions	• *“It is quite common that they [doctors] use unsterile gloves for the wound dressings and they believe it’s sufficient. It is different from what we [nurses] learned in our education. I find it difficult to argue. I have never experienced a wound has worsen because of that. But I ask myself why do I take gloves, and they …don’t? What is the evidence then?“* Nurse, ward (X215)
Anticipation of negative outcomes (for the patient, the actor, and themselves)	• *“Because I would have had shown her [the actor, doctor] up, and the patient would become anxious then. I wanted to avoid that”.* Nurse, ambulatory unit (C113)
	• *“To me, the risk that something severe happens is considerably lower than the fact that the chief’s authority is questioned in front of the patient. This harm is considerably higher than not disinfecting hands once”.* Senior, ward (B102)
	• *“I feel that, if it would be really important for the patient, then I speak up. And I would even speak up to the director, but it would cost me quite an effort”.* Nurse, pediatric ward (J224)
	• *“I rather prefer to risk hassle; that she [the actor, senior doctor] would not respond adequately and would be angry with me…I would rather hazard these consequences than doing something I’m not fully convinced of or doing something which I know is not correct”.* Resident, ward (J118)
Predictability of the actor’s response	• *“In our team, you can forecast how the response will be. You know how people react, what people can accept and what not. With someone you have only seen once or twice, you do not know that”.* Nurse, ambulatory unit (P225)
	• *“Because residents alternate so frequently, relations are not so much established and we do not have the trust in them, and they probably not in us”.* Nurse, ward (B205)
	• *“With the surgeons it is difficult in particular. I have known our oncologists for years now and I know how they will respond to my speaking up. With doctors I see once a year, I find it difficult to intervene”.* Nurse, ward (X215)

As most important component, the potential of patient harm associated with an error or rule violation was assessed and entered the “calculation”. While the motivation to prevent patient harm was overarching, a third of professionals elaborated extensively on the relevance of the *level of risk* for their decision to speak up. Respondents seemed to have systematically lower thresholds for voicing medication safety concerns, compared to other issues. In particular, violations of hygiene standards were usually not perceived as justifying speaking up. Some interviewees explicitly referred to differences in judgments of potential of patient harm, in particular, between doctors and nurses. On the other side of the calculation, participants tried to anticipate possible negative outcomes for the patient, the actor, and themselves.

For the calculation of potential costs of speaking up, anticipating the actor’s response is prerequisite. The “predictability” of the actor’s response to participants’ voicing behaviours was an important issue for nearly half of interviewees. Speaking up to well-known co-workers was described as considerably easier whereas “not knowing the actor well” increases risks and potential costs of speaking up. Two constellations were perceived as particularly difficult: Residents in oncology rotation and specialists from other departments (e.g., surgeons or anaesthesiologists). Participants’ reports indicated that it was the lack of predictability of the outcome and thus “uncontrollability” that affected their willingness to speak up.

### Conceptualizing the decision to speak up

**Figure 1:**
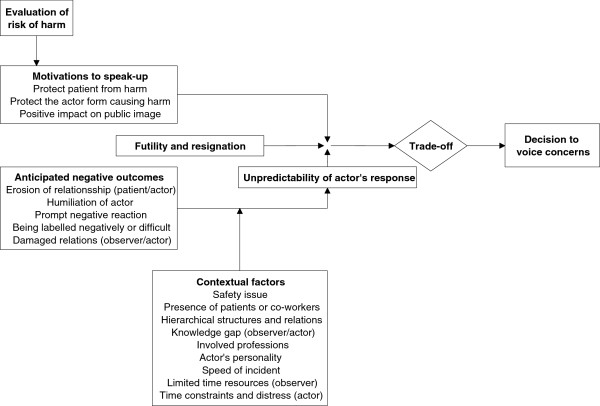
Conceptual model of the decision to voice concerns.

We conceptualize the contributions of motivations and barriers to the decision to speak up as displayed in Figure 1. The strength of the motivation to voice concerns is based on an evaluation of the potential harm for the patient. As noted above, clinicians described detailed risk assessments following their co-workers rule violations. The higher the potential for harm, the stronger (ceteris paribus) the motivation to speak up. The perceived benefit of speaking up (i.e. preventing harm) is countered by a variety of fears related to anticipated negative outcomes. These fears relate to the patient, the actor, and the self. Despite anticipated negative outcomes, futility and resignation decrease perceived effectiveness of speaking up, i.e., the perception that voicing concerns will not make a difference (often experience-based) and thus the likelihood of action. A number of contextual factors, such as the presence of patients and co-workers in the alarming situation, affect the likelihood and severity of anticipated negative outcomes. Several personal accounts of interviewees suggest that many of these contextual factors act as interacting variables (moderators/mediators). For example, the strength of the link between hierarchical status of the actor and anticipated negative outcomes of speaking up is probably moderated by safety issue to be voiced. The unpredictability of the actor’s response limits feelings of control and is thus conductive of "silence". Though this factor can be regarded “contextual” it is of special character as it affects the *predictability* of outcomes and thereby the entire trade-off and decision making process.

## Discussion

In this study we explored motivations and barriers to speaking up perceived by doctors and nurses in oncology. To the best of our knowledge, this is the first study which assessed speaking up behaviors in the context of cancer care. Participants reported many situations in which they felt comfortable to voice their safety concerns towards co-workers and supervisors. However, episodes of remaining silent were a common experience among staff and were reported by all professions and hierarchical levels. The fundamental motivation for speaking up was to protect individual, identifiable patients from harm. While doctors and nurses felt strong obligation to prevent errors and safety violations reaching the patient, they were not engaged in voicing concerns beyond this immediacy. Preventing future harm in “statistical patients”, improving safety systems, or contributing to a learning organization were not mentioned as motives to voice concerns. This is exemplified by episodes in which observers could not stop the error or questionable behavior in due time. Only rarely had the observers taken opportunity to discuss the event later. Thus, actors in these episodes did not receive feedback on their performance. This is an important and novel finding. From a systems and learning perspective situations in which "statistical, future patient lives" are at stake and threatened by risky behaviours, may be even more relevant. Even if a clinical error cannot be prevented from reaching an individual patient, speaking up post-hoc would be important to avoid the error recurring and to open opportunities for learning and changing behaviour. Further research is needed into low-threshold interventions that would facilitate post-hoc speaking up behaviours, such as after action reviews or medication event huddles [27,28].

While our study confirms many of the facilitating and impeding factors affecting voicing behavior reported in previous research, we also report some novel findings which may be characteristic for oncology and similar areas of clinical care. Staff reported various barriers for voicing concerns and weighted anticipated benefits against negative outcomes, e.g., damaged relationships or humiliation of a co-worker. In particular, many respondents reflected in detail on whether the level of risk for a patient “justifies” speaking up and its associated costs. Questions and concerns relating to medication safety were much less likely to remain unexpressed compared to violation of safety rules, issues related to hygiene and isolation. Also, staff was highly concerned with damaging (often long and intense) patient-provider relationships, trust of patients, and putting additional burden on the severely ill, and this was a strong motivation to withhold voice whenever patients or family were present. Whether this motivation is as strong outside oncology has yet to be confirmed. Obviously, there exists an imaginary threshold of potential harm (likelihood and severity) which enters the “internal calculus” whether and how to voice concerns. Of note, many individuals were well aware of the trade-offs they make. It is concerning that nurses and doctors *believe* that they are able – often within a heartbeat – to accurately estimate the risks associated with a specific behavior in a specific patient, e.g., not using gloves for lumbar puncture in a child. This “relative” interpretation of safety rules caused dissonance in some oncology nurses and resulted in feelings of resignation and futility often termed as “acquiescent silence” in the organizational silence literature [4]. We suggest that nurses and residents need to be encouraged by unit leaders to defend these safety rules. Importantly, supervisor’s attitudes to silence, and thus the micro-climate, are a key predictor of employees silence behavior [29].

Nembhard and Edmondson recently introduced the concept of leadership inclusiveness, which describes “words and deeds exhibited by leaders that invite and appreciate others' contributions”, including speaking-up [30]. Based on survey data obtained in neonatology they report that leader inclusiveness predicts staff psychological safety, an important antecedent of speaking-up. This stresses supervisors’ role in establishing and reinforcing a “culture of voice” [31]. The unambiguous safeguarding by leaders is of significance if resident rotation and frequent co-operation with external specialities makes the predictability of consequences of speaking up difficult.

The relevance of fears relating to damaging social relationships and being labeled negatively is supported by previous research both inside and outside healthcare [9,13,32]. However, in our study anticipated negative effects on the relation between patient and the actor were even more critical barriers to voicing concerns. The presence of patients, relatives and other co-workers during the error or violation made the trade-off between speaking up and remaining silent exceptionally difficult. Participants often reported using questions, gestures and non-verbal signals to point the actor to the problem, but this communication was not always successful. More research is clearly needed to identify voicing strategies that would be acceptable to observers and actors in such situations. For example, teaching anaesthesia residents the two-challenge rule, a conversational technique that is assertive (advocacy) and collaborative (inquiry), increased frequency and effectiveness of challenges towards other physicians in operation room simulations [33]. Such instructional interventions could be transferred to settings outside the operating room and seem particularly valuable for care teams involving different professions, specialities, and hierarchies, like oncology. Based on the findings of our study, we can clearly conceptualize scenarios with high and low difficulty of speaking up. These scenarios could be used to discuss and train speaking up behavior with oncology staff. They could also be embedded in a survey study as vignette case stories to estimate the relative, quantitative importance of different factors. More generally, the results of our study could be helpful to develop health care specific survey measures to assess trade-offs in voicing decisions.

Our study generally confirms existing models of employee voicing behaviors [4,13] but also suggests some refinements and adjustments. In particular, we suggest some important contextual variables which are likely to have moderating and mediating effects in the health care setting (e.g., the presence of patients and family). The role and prevalence of perceived unpredictability of speaking up situations as a result from less known co-workers warrants further study. Our extended model of voice and silence can be used to generate hypotheses and quantitative testing.

### Limitations

The main limitation of our study is associated with the design: Our findings are based on clinicians’ reports of motivations and barriers. As such, they rely on participants’ introspection and are subject to various biases. For example, clinicians’ judgments of “low harm” associated with others’ behaviors may result from post-hoc rationalizations of their own failures to speak up rather than from concurrent risk assessments. Ethnographic observation would be an alternative to study speaking up behaviors in the field. However, the effects of contextual factors on HCPs’ voicing behaviours are not easily accessible and controllable in observational studies. As Tangirala argues, “silence” is a nonbehavior and as such difficult to observe, and even more difficult to interpret [34]. While qualitative observation is a strong and valuable method to gain an in-depth understanding of HCPs’ real behaviours, it is limited to actual occasions and, by definition, cannot be extended to answer 'what if' questions. We propose future research to employ a triangulation of methods, including qualitative observation and quantitative surveys under experimental designs. Also, the extension of simulation studies to outside the operating room would be highly valuable.

## Conclusions

HCPs in our study were often comfortable to voice their safety concerns towards co-workers and supervisors but also experienced multiple barriers. In particular concerning medication safety, there exists a well-established culture that expression of questions and concerns is expected and desired. Preventing cancer patients from harm is the dominating motivation for voicing concerns even if speaking up is perceived as dangerous. However, clinicians need to be made aware of the importance and value of speaking up beyond the particular situation and the current patient involved. Our results offer in-depth insight into fears and conditions conducive of silence and voicing and can be used for educational interventions and leader reinforcement.

## Competing interests

The authors declare that they have no competing interests.

## Authors’ contributions

Both authors made significant contributions to study conception and design, coordination, and data analysis and interpretation. DS drafted the manuscript. KG revised it critically for important intellectual content. Both authors read and approved the final manuscript.

## Pre-publication history

The pre-publication history for this paper can be accessed here:

http://www.biomedcentral.com/1472-6963/14/303/prepub
